# Dietetic Treatment of Nutritive Disorders in Children

**Published:** 1884-12

**Authors:** 


### Dietetic Treatment of Nutritive Disorders in Children.

Dr. Bidert has treated a number of cases of infantile digestive disorders without drugs, by means of a strict regulation of the diet. The diseases treated were dyspepsia, dyspeptic diarrhœa, chronic catarrh, extreme atrophy (tabes meseraica), ulcerative enteritis, cholera infantum, and one supposed case of epidemic dysentery. The children were most carefully watched, and the greatest care observed in carrying out the minute details of treatment. From the results obtained the author feels himself justified in recording the following deductions (*Centralblatt für Klinische Medizin*): 1. A surprisingly large number of gastro-intestinal disorders in infants stand in such close relation with the quality and insufficient quantity of food that the diseases, even in the most serious cases, may be cured solely by the administration of a suitable diet. 2. The quantity of food given is of the greatest moment. 3. The nourishment must often be given in greatly diluted form. 4. The proportion of albumen to fat plays an important role. The digestion of albumen is facilitated by mixing it with a much larger proportion of emulsionized fat than is found in cows' milk—that contained in human milk being the proper amount. 5. It should not be forgotten that, at times, there is a diminished absorption of fat, in which case it should be greatly reduced in amount, or, in order not to interfere with the digestion of albumen, slightly reduced to a proportion midway between that of human milk and cows' milk.—*Medical Record.*

